# Dataset of shotgun metagenomic evaluation of lettuce (*Lactuta sativa* L.) rhizosphere microbiome

**DOI:** 10.1016/j.dib.2023.109214

**Published:** 2023-05-09

**Authors:** Olubukola Oluranti Babalola, Akinlolu Olalekan Akanmu, Ayomide Emmanuel Fadiji

**Affiliations:** Food Security and Safety Focus Area, Faculty of Natural and Agricultural Sciences, North-West University, Private Bag X2046, Mmabatho, South Africa.

**Keywords:** Shotgun approach, Microbial community, Functional genes, Root microbiomes, SEED subsystem, Illumina, Lettuce metagenome, MG-RAST

## Abstract

Lettuce (*Lactuca sativa* L.) is an important vegetable grown and consumed across the world, including South Africa and its rhizosphere constitutes a dynamic community of root associated microbes. Dataset of the microbial community profile of the lettuce rhizospheric soils obtained from Talton, Gauteng Province of South Africa was subjected to metagenomic evaluation using the shotgun approach. The whole DNA isolated from the community was sequenced using NovaSeq 6000 system (Illumina). The raw data obtained consists of 129,063,513.33 sequences with an average length of 200 base pairs and 60.6% Guanine + Cytosine content. The metagenome data has been deposited to the National Centre for Biotechnology Information SRA under the bioproject number PRJNA763048. The downstream analysis alongside taxonomical annotation carried out using an online server MG-RAST, showed the community analysis as being made up of archaea (0.95%), eukaryotes (1.36%), viruses (0.04%), while 97.65% of the sequences were classified as bacteria. A sum of 25 bacteria, 20 eukaryotic and 4 archaea phyla were identified. The predominant genera were *Acinetobacter* (4.85%), *Pseudomonas* (3.41%), *Streptomyces* (2.79%), *Candidatus solibacter* (1.93%), *Burkholderia* (1.65%), *Bradyrhizobium* (1.51%) and *Mycobacterium* (1.31%). Annotation using Cluster of Orthologous Group (COG) showed 23.91% of the sequenced data were for metabolic function, 33.08% for chemical process and signaling while 6.42% were poorly characterized. Furthermore, the subsystem annotation method showed that sequences were majorly associated with carbohydrates (12.86%), clustering-based subsystems (12.68%), and genes coding for amino acids and derivatives (10.04%), all of which could serve in growth promotion and plant management.

**Specifications Table**

**Table:** 

Subject	Microbiology

Specific subject area	Microbiome
Type of data	Figures and Raw NGS data (Fastq files)
How the data were acquired	Four lettuce varieties (Green Oak, Red Frilly, Salanova Green and Welton) rhizospheric soils, and the bulk soil (control) were sampled at 3 replicates each (a total of 15 samples). DNA from soil samples were extracted and sequenced on Novaseq 6000 platform (2×150 paired end). Raw data were assembled and annotated through online MG-RAST service.
Data format	Raw data (fasq.gz.file)
Description of data collection	Metagenomic DNA were extracted from rhizospheric soils samples of four lettuce varieties and bulk (control) from Gauteng Province, South Africa. NucleoSpin Soil kit (Macherey-Nagel, Germany) was used for DNA extraction, NGS on NovaSeq 6000 system (Illumina) and metagenome sequence annotation were carried out using MG-RAST.
Data source location	Institution: North-West University, Mmabatho, North West Province, South Africa. Latitude and longitude for the collected soil samples: Talton, Gauteng Province, South Africa (26°05’14.9"S 27°37’08.6"E). Latitude and longitude for the collected bulk soil samples: (26°08′16.9"S 27°37′04.9"E).
Data accessibility	Repository name: National Centre for Biotechnology Information SRA **Data Identification Number:** PRJNA763048 **URL:**https://www.ncbi.nlm.nih.gov/bioproject/PRJNA763048[1]

## Value of the Data

• The dataset provides information on the abundance, diversities and functions of microbes associated with lettuce varieties.
• It indicates the influence and peculiarities of each lettuce variety on the selection of microbes around the plants’ roots.
• The dataset can provide further insight on the influence of varieties in plant-microbe interactions and can be employed as genetic and molecular basis for host resistance.
• This dataset provides preliminary insights into the possibly untapped roles of the culturable and unculturable rhizospheric microbes.
• The dataset provides the prospects of finding novel genes and plant-growth promoting compounds that could enhance lettuce improvement and cultivation.

## 1 Objective

The advent of New-Generation Sequencing (NGS) technology, which is inclusive of metagenomics analysis has enabled a deeper insight into the understanding of the composition and function of soil microbial communities. Therefore, this dataset unveils the community structure and functional diversity of rhizospheric microorganisms associated with roots of four lettuce varieties using a shotgun metagenomics approach.

## 2 Data Description

**Fig. 1:**
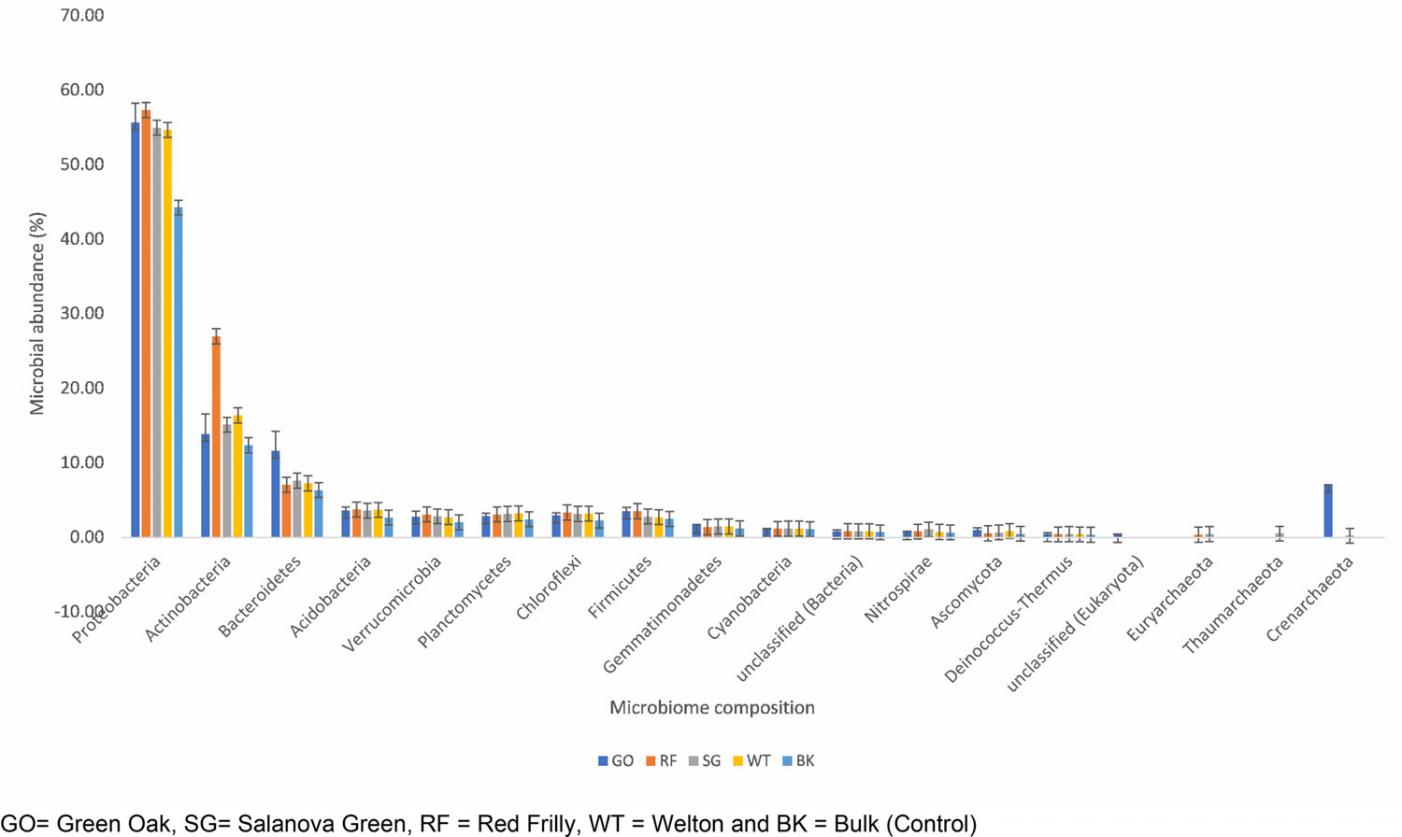
Phyla obtained according to the taxonomic annotation of lettuce rhizosphere microbiome Each bar represents the mean ± standard error of each phyla component recovered.

**Fig. 2:**
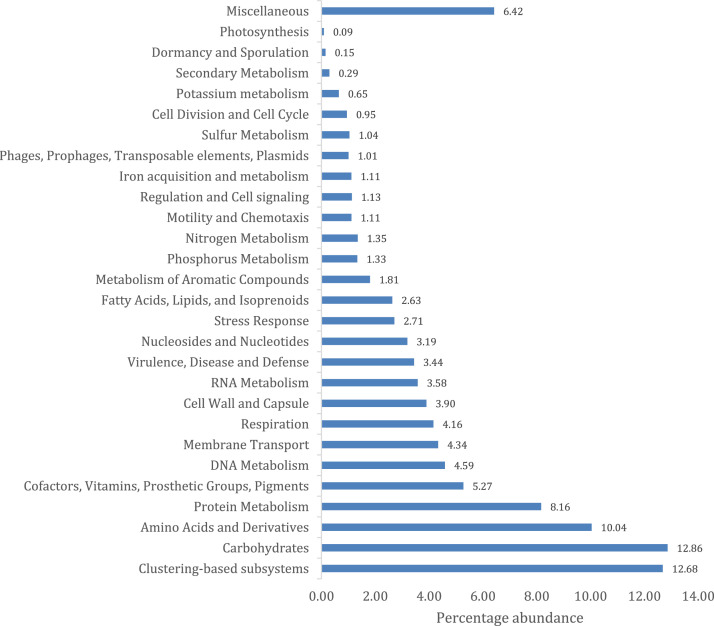
Combined functional profiles of lettuce rhizosphere microbiome dataset using SEED subsystem.

The dataset is made up of raw NGS data obtained through shotgun sequencing of lettuce rhizosphere metagenome. All the obtained datasets in fastq.gz file was deposited at the NCBI SRA database (PRJNA763048). Details of the microbial community and functional structure determined using SEED subsystem were shown in Fig. 1, Fig. 2 respectively.

GO= Green Oak, SG= Salanova Green, RF = Red Frilly, WT = Welton and BK = Bulk (Control)

## 3 Experimental Design, Materials and Methods

Rhizosphere soil samples were collected from a cultivated lettuce (*Lactuta sativa* L.) field in Gauteng Province, South Africa (26°05’14.9"S 27°37’08.6"E, altitude, 159 km). As earlier described [2,3], soil tightly-bound to plant root were scraped from each of the four lettuce varieties; Green Oak, Red Frilly, Salanova Green and Welton, while the bulk soil (control) was collected from a natural grassland 20 m away from the lettuce plantation. Using a calibrated scale, 500 mg of each collected soil samples was weighed while extraction of the whole community DNA was carried out using the NucleoSpin Soil kit (Macherey-Nagel, Germany) according to the manufacturers’ guidelines. Shotgun metagenomic sequencing was carried out using NovaSeq 6000 system (Novogene, Singapore). The default specification of online server Metagenomic Rapid Annotation Subsystem (MG-RAST) [4] was used for the structural analysis and functional annotation of the sequenced data. After quality assessment, sequenced data were annotated using a BLAST-like alignment algorithm called BLAT [5], against M5NR database [6] which offers concise alliance with many other databases.

## CRediT authorship contribution statement

**Olubukola Oluranti Babalola:** Conceptualization, Supervision, Software, Validation, Resources, Writing – review & editing, Project administration, Funding acquisition. **Akinlolu Olalekan Akanmu:** Methodology, Investigation, Software, Formal analysis, Data curation, Writing – original draft, Writing – review & editing. **Ayomide Emmanuel Fadiji:** Methodology, Visualization, Investigation, Software, Writing – review & editing.

## Declaration of Competing Interest

The authors declare that they have no known competing financial interests or personal relationships that could have appeared to influence the work reported in this paper.

## Data Availability

Metagenome of Lettuce Rhizosphere in Gauteng South Africa (Original data) (NCBI_SRA)
